# Understanding and harnessing triple-negative breast cancer-related microbiota in oncology

**DOI:** 10.3389/fonc.2022.1020121

**Published:** 2022-11-24

**Authors:** Ciaran Devoy, Yensi Flores Bueso, Mark Tangney

**Affiliations:** ^1^ Cancer Research@UCC, College of Medicine and Health, University College Cork, Cork, Ireland; ^2^ SynBio Center, University College Cork, Cork, Ireland; ^3^ APC Microbiome Ireland, University College Cork, Cork, Ireland; ^4^ School of Pharmacy, College of Medicine and Health, University College Cork, Cork, Ireland

**Keywords:** microbiome, triple-negative-breast-cancer, microbiome & dysbiosis, bacterial theranostics, microbiome modulation

## Abstract

Bacterial inhabitants of the body have the potential to play a role in various stages of cancer initiation, progression, and treatment. These bacteria may be distal to the primary tumour, such as gut microbiota, or local to the tissue, before or after tumour growth. Breast cancer is well studied in this context. Amongst breast cancer types, Triple Negative Breast Cancer (TNBC) is more aggressive, has fewer treatment options than receptor-positive breast cancers, has an overall worse prognosis and higher rates of reoccurrence. Thus, an in-depth understanding of the bacterial influence on TNBC progression and treatment is of high value. In this regard, the Gut Microbiota (GM) can be involved in various stages of tumour progression. It may suppress or promote carcinogenesis through the release of carcinogenic metabolites, sustenance of proinflammatory environments and/or the promotion of epigenetic changes in our genome. It can also mediate metastasis and reoccurrence through interactions with the immune system and has been recently shown to influence chemo-, radio-, and immune-therapies. Furthermore, bacteria have also been found to reside in normal and malignant breast tissue. Several studies have now described the breast and breast tumour microbiome, with the tumour microbiota of TNBC having the least taxonomic diversity among all breast cancer types. Here, specific conditions of the tumour microenvironment (TME) - low O2, leaky vasculature and immune suppression - are supportive of tumour selective bacterial growth. This innate bacterial ability could enable their use as delivery agents for various therapeutics or as diagnostics. This review aims to examine the current knowledge on bacterial relevance to TNBC and potential uses while examining some of the remaining unanswered questions regarding mechanisms underpinning observed effects.

## 1 Introduction

Triple-Negative Breast Cancers (TNBC) characteristically lack, or express at very low levels, human growth factor receptor 2 (HER2), progesterone (PR) and/or estrogen receptors (ER) (1). In 2020, 12% -17% of the 2.3 million new breast cancer (BC) cases and over 685,000 deaths worldwide can be attributed to TNBC (2). TNBC disproportionally affects young premenopausal women with west African ancestry, particularly African American and Ghanaian women (3). Other relevant risk factors include Breast Cancer gene-1/2 (BRCA) mutations, smoking history, and obesity (4, 5). Generally, TNBC originates in the milk duct as ductal carcinoma and less frequently in mammary lobules as lobule carcinoma (6). Based on its genotype profiling and cellular origin, TNBC can be classified into four subtypes: basal subtypes 1/2, mesenchymal subtype and an androgen receptor-expressing luminal subtype (7).

Surgery (lumpectomy or mastectomy) followed by radiation is available for early-stage patients, and immune checkpoint inhibitor therapies are offered on a case-by-case basis (8). For those that have missed the surgical window, the standard treatment for non-metastatic TNBC at the early stage is still nonspecific chemotherapy including platinum, taxane, anthracycline and cyclophosphamide with checkpoint inhibitor immunotherapy, such as atezolizumab or pembrolizumab given as a neoadjuvant where tumors are greater than 2cm in diameter and lymph node-positive (9). Adjuvant capecitabine treatment is standard in the case of residual disease in conjunction with PARP inhibitors olaparib or talazoparib, where BRCA-1/2 mutations are present (10). TNBC is highly invasive and has no standard treatment care options for the metastatic disease stage. Therapeutic schemes for this disease are constrained to conventional cytotoxic chemotherapy with additional immunotherapy targeting programmed death receptor 1 or ligand (PD-1 or PDL-1), as endocrine or receptor based therapies (e.g. HER2, ER and PR) are completely ineffective for TNBC (11). Recently antibody drug conjugates such as sacituzumab govitecan have been approved for metastatic TNBC (12).

## 2 The human microbiome and cancer

Humans are a symbiont of human and microbial cells, with a ratio of 1.3-2.5 bacterial: human cells (13). Although the majority of these microbes reside within our gut, distinctive collections of microbes are also found in most body parts, possibly even including the brain, although present evidence is inconclusive (14). These distinctive microbial signatures are known as *the microbiome* (15, 16). The term microbiome, as Whipps and co-workers originally postulated, includes not only the community of the microorganisms but also their “theatre of activity”. These ecosystems, created by a multitude of microbes that may include bacteria, archaea, fungi, yeast, and viruses, are site-specific (15). The microbiome of each body part has distinctive characteristics regarding population dynamics and the diversity of microbial species (17). This site-specific diversity and dynamics can be regarded as a health indicator with, high diversity in the gut microbiome generally linked to good health (18). Our microbiomes represent a virtual organ that performs essential body functions that maintain our homeostasis, such as metabolizing nutrients, maintaining the integrity of the mucosal barriers, developing a healthy immune system, modulating a healthy neuronal development (including regulating our moods) and defending us against pathogens (19–21).

### 2.1 Distal (gut) microbiome and cancer

#### 2.1.1 GM composition and dysbiosis

Microbes start colonizing our body as early as in the 2^nd^ trimester of fetal development, where low levels of microbial signals can be detected in the fetal gut, skin, placenta, and lung tissue (22). However, the first major colonization event in early life happens at birth, where the mode of delivery determines the neonate microbiome composition to resemble either a vaginal or skin microbiome (23). After this event, our microbiomes are shaped by external factors such as diet, lifestyle, and environmental biodiversity (24). Our microbiome composition varies with age. In early neonatal life, breastfeeding enables the vertical transmission (mother to infant) of bacteria. Thus, neonates exhibit a microbiome composition resembling their mother’s milk. The adult type of GM composition starts appearing at ages 3-5 years (25). At this age, 90% of all species of the adult GM would have already colonized the gut.

**Figure 1:**
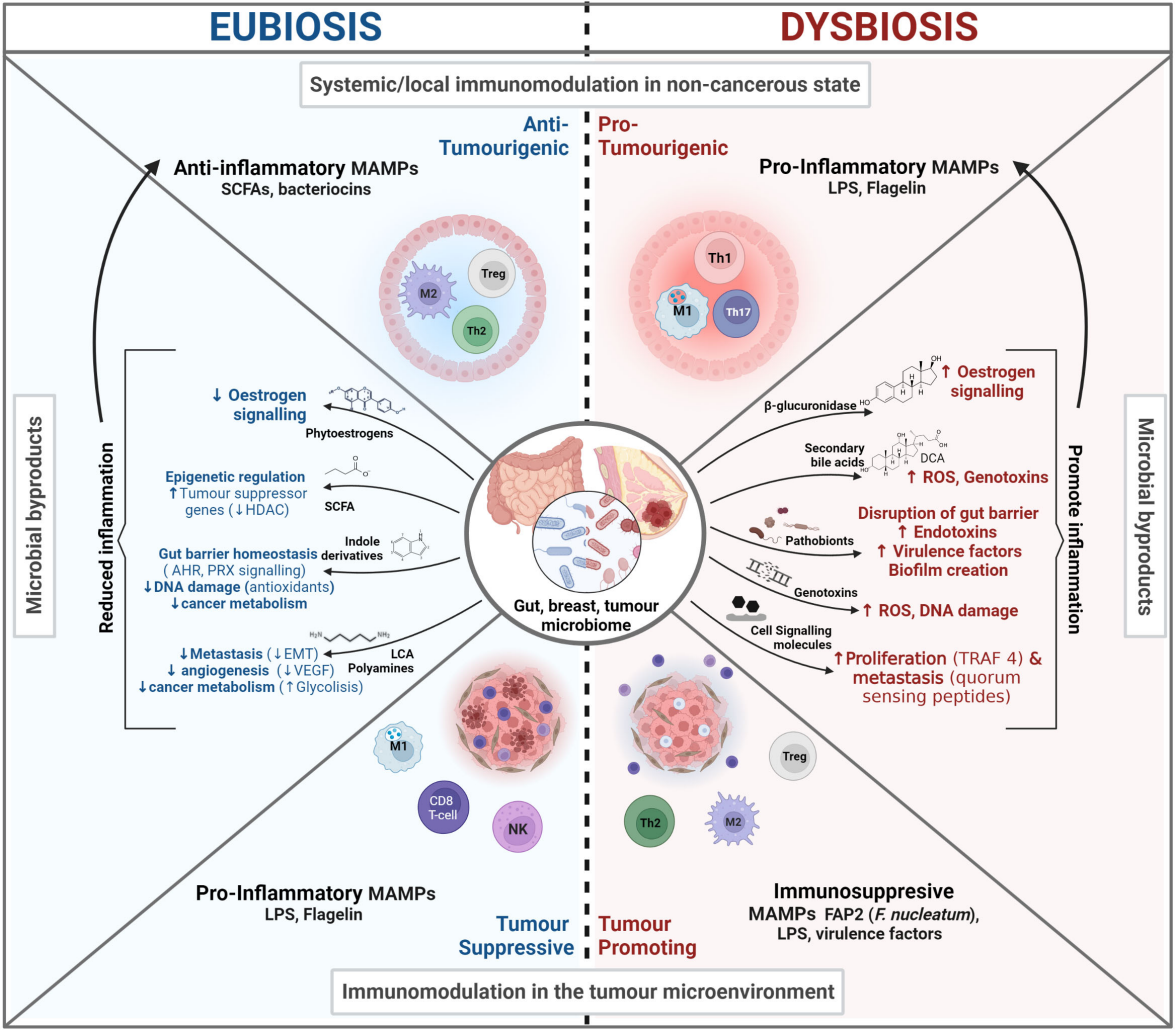
The microbiome influence in Cancer. In eubiosis the microbiome promotes our health status and prevents oncogenesis and tumor progression by influencing the immune system, promoting gut barrier integrity, and influencing cell signaling. Conversely, during dysbiosis there is a loss of barrier integrity that can lead to potentially harmful bacterial translocation, chronic inflammation in distal sites and the production of cancer promoting bacterial by-products.

Overall, a diverse GM is a healthy and robust GM, well able to perform the multiple tasks that define our health status (see **Figure 1**) (26). On average, the adult GM is estimated to be composed of 300-500 species (27), comprised of 12 bacterial phyla and one Archaean taxon, with the majority of species belonging to the Bacteroidetes and Firmicutes phyla and a smaller proportion to the Proteo- and Actinobacteria phyla (28, 29). However, GM composition differs between individuals and starting in mid-to-late adulthood (40-50 years of age), it increasingly diverges towards a microbiome that is unique to each individual (28, 30). Compositional uniqueness is more accentuated among the elderly (>65 years old) since, at this life stage, microbiome diversity can decrease significantly. In this age group, uniqueness has been positively associated with a healthy status (24, 30).

A healthy microbiome safeguards host-microbiome homeostasis. Here, different microbe populations regulate the abundance of neighboring commensal or pathogenic bacteria by occupying a niche and adjusting the niche environment. Bacteria compete for nutrients, release bacteriocins (peptides which are toxic to and inhibit/regulate the growth of similar or closely related bacterial strains) or bacterial signals (MAMPs, see 1.2) to communicate with the host in order to modulate the release of antimicrobial peptides, mucin and ultimately, immune responses (31). Together, these actions contribute to the formation and maintenance of a healthy GIT mucosal “firewall”, which by segregating the GM from host cells, prevents microbial translocation and adverse immune priming events (32, 33).

Overall, a healthy GM safeguards host-microbiome homeostasis by modulating immune tolerance against gut commensals and eliciting pertinent immune responses (34). These GM-immune cell interactions are essential for the proper development of the gut-associated lymphoid tissues (GALT), which is the largest mass of lymphoid tissue in the body (more in 1.2) (35). The loss of beneficial microbes, expansion of pathobionts (commensals that at higher densities can cause harm), and/or the overall loss of microbial diversity can alter the GM composition in a way in which the abovementioned self-regulation and host-microbiome homeostasis functions are impaired. This altered and impaired GM composition is known as dysbiosis (36). GM Dysbiosis has been found to influence tumorigenesis through multiple mechanisms and interactions, including modulating our immune system, the metabolism of estrogens, and the production of protective or oncogenic metabolites, as described in **Figure 1** and sections 2.1.3-2.1.4 (32).

#### 2.1.2 The GM and immunity

**Figure 2:**
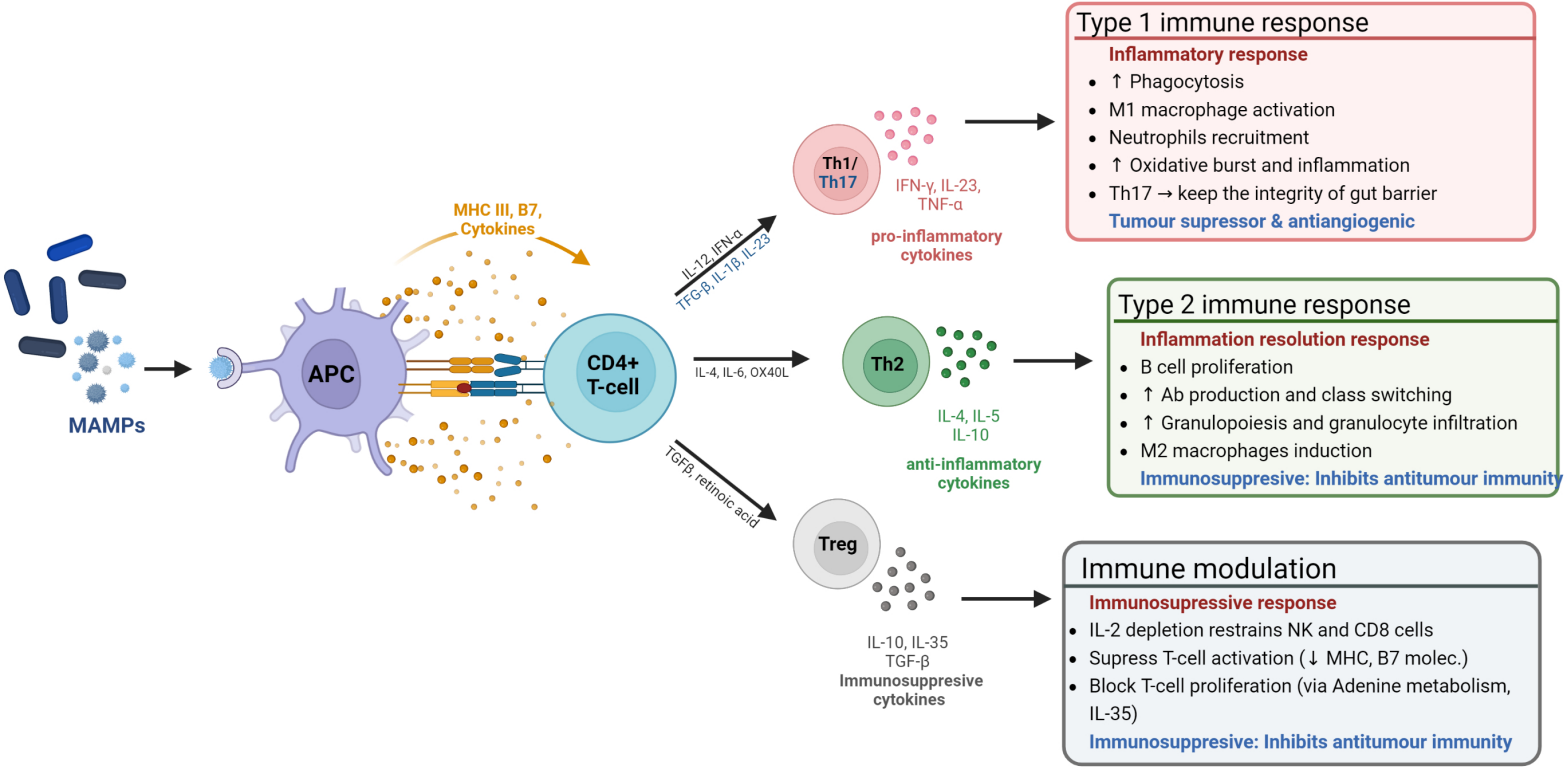
The microbiome influence on the host immune system. The microbiome modulates the immune system through MAMP production. These are sensed by antigen presenting cells (APCs), which process them to be presented to CD4+ naïve T-cells to induce either immune tolerance or an immune response.

The microbiome promotes the development and maintenance of the GIT mucosa and associated lymphoid tissues by producing microbial motifs (antigens) known as microbe-associated molecular patterns (MAMPs) (e.g., lipopolysaccharide (LPS), short-chain fatty acids (SFCA), and peptidoglycans) (37–39). The more commonly known PAMPs - pathogen-associated molecular patterns - are a subset of MAMPs, which include non-pathogenic microbes. MAMPs are recognized by pattern recognition receptors (PRRs) in Antigen Presenting Cells (APCs)). The GM uses MAMPs to communicate, trains and supports the maturation of the innate immune system, in order to: *(i)* modulate tolerance by discerning self from non-self (40); *(ii)* ensure homeostatic levels of innate immune cells (e.g. macrophages and dendritic cells) (41); *(iii)* bridge the innate and adaptative immune systems, through the production of co-stimulatory signals that induce an adaptive immune system response (42, 43). See **Figure 2**.

This is particularly relevant for TNBC, wherein the tumor microenvironment is characteristically immunosuppressive, featuring immunosuppressive and pro-metastatic cell types and factors, such as: Type 2 macrophages (M2) and neutrophils (N2), Cancer-Associated Fibroblasts (CAFs), Cancer-Associated Adipocytes (CAAs), and altered extracellular matrix (ECM) (44). In fact, a current TNBC clinical trial (NCT02981303) is attempting to harness these TNBC immunosuppressive features and the immunostimulatory effects of PAMPs, by using a soluble yeast β-1,3/1,6-glucan PAMP (referred to as Imprime), to enhance the effect of immunotherapies after positive pre-clinical trials (45).

#### 2.1.3 GM influence in TNBC *via* chronic inflammation

Chronic inflammation is a long-term reaction over the course of weeks or even an entire lifetime to inflammatory stimuli with continuous recruitment of monocytes and lymphocytes in addition to local tissue damage caused by the prolonged inflammatory response itself (46). Chronic inflammation contributes to tumorigenesis at all stages of oncogenesis, progression and dissemination, by promoting genomic instability and epigenetic modifications, inducing proliferation, strengthening anti-apoptotic pathways, stimulating angiogenesis and metastasis (47, 48). Roughly 10% of cancers are a result of a non-modifiable factors such as genetic predisposition to tumor development, with the remaining 90% a result of modifiable factors which induce DNA damage through environmental or life-style factors, for example U.V. damage, smoking, diet, alcohol use, obesity and infection status, that directly damage DNA or lead to some form of chronic inflammation (49). Among these, up to 20% of all cancers develop in tissues commonly affected by chronic inflammation (50).

Inflammation is a mechanism mainly orchestrated by the innate immune system to defend the body against pathogens and injuries, which can be perpetuated by exogenous stimuli (e.g. MAMPs) (51, 52). The prolonged/recurrent exposure to stimuli can lead to an uncontrolled infiltration and accumulation of immune cells, which can become polarized towards pro-inflammatory, tumor-promoting cell types (Type 1/17, 2 in **Figure 2**). It can also lead to the release/production of damage-associated molecular patterns (DAMPs), which signal a status of altered-self and amplify and perpetuate immune reactions through a vicious cycle of inflammation and DAMP production (53, 54).

The most relevant mechanism for bacterial-driven chronic inflammation is gut-barrier failure (33, 55, 56). A healthy barrier prevents the translocation of microbes into compartments where they would elicit a systemic immune response and establish immune memory (57). This anatomical separation is achieved through a multi-level gut-barrier, which ensures (*i) segregation*: preventing direct contact between the GM and host tissue; and (*ii) compartmentalization* – ensuring that responses to commensal bacteria are kept locally (56). The disruption of segregation/compartmentalization can lead to leaky-gut syndrome: A change in the permeability of the gut epithelial lining enabling the translocation of microbes or their by-products from the gut-lumen into the bloodstream, where they can be distributed systemically to other body sites and incite inflammatory responses (58). The recurrent injury to the tissue caused by these responses can consequentially prime the immune system (*via* DAMPs) towards chronic inflammation (59). In fact, a commonality across GM-associated diseases is that they are all a product of barrier disfunction (50, 60). This has been correlated to localized digestive tract diseases, such as in colon (61), liver (62) and pancreatic cancer (63), among others; and systemic inflammatory diseases, such as metabolic (64) autoimmune diseases (65), and cancer in other body sites (32), including the breast (66). A study looking at the inflammatory pathway score using a defined set of inflammatory genes from 3,632 tumors from four BC cohorts came to the conclusion that inflammation was associated with worse outcome overall in the BC cohort, but in TNBC, was associated more positively with tumor clearing immune response and immune cell infiltrations (67).

#### 2.1.4 GM influence in TNBC *via* GM by-products

The GM produces a large array of small molecules during the metabolism of food and xenobiotics (compounds of nonhost origin that enter the gut with the diet, lifestyle or are produced by microbes). These can be in the form of low molecular weight metabolites, peptides, and proteins. In fact, the GM is associated with many biochemical pathways and in the synthesis of specific metabolites that are absorbed into the circulation. In this way, the GM contributes to the host biology a circulating pool of bacterially derived metabolites that can potentially exceed concentrations typically achieved by drugs (10 μM–1 mM) (68). Many of these by-products play critical roles in interbacterial (between different microbial species) and host-GM signaling by engaging with specific host receptors (69). As such, GM-derived metabolites can signal distant organs in the body and facilitate a connection between the host immune and hormone system, brain (the gut-brain axis), and metabolism. The beneficial or detrimental effects of these GM-derived metabolites will depend on the context and the state of the host, considering that the primordial nature of the symbiotic microbiota is to ensure its host health (70). Accordingly, below are outlined some of the pathways, metabolites and/or by-products by which the GM can exert an effect in TNBC.

##### 2.1.4.1 GM influence on estrogen regulation

The risk for developing receptor-positive breast cancers in post-menopausal women is highly associated with the levels of circulating estrogens and the time of exposure (71, 72). Whilst estrogen metabolism is not traditionally considered an essential factor in TNBC, it may be to a degree, as some TNBCs express alternative estrogen receptors. In addition, different jurisdictions have different cut-offs for the expression of either estrogen receptor (ER) and/or progesterone receptor (PR). For example, in the US, TNBC is diagnosed when receptor expression is lower than 1%. On the other hand, in the European Union, TNBC is diagnosed when the expression levels are lower than 10%.

Furthermore, estrogen signaling in TNBC can also be maintained by constitutively active estrogen-related receptors (ERRs) other than the canonical ER-α (73). Among these, estrogen receptors ERβ and the G protein-coupled estrogen receptor 1 (GPER-1) enable some degree of estrogen reactivity in TNBC (74). While the role of GPER in cancer is still inconclusive, new evidence shows that ER-β can have anticancer effects, including for TNBC. For example, a recent study looking into a cohort of 567 TNBC tumors, found that ERβ was expressed in 18% of them. Possible mechanisms for ERβ mediated tumor suppression include the formation of co-repressor complexes that suppress the activity of oncogenic NFκB/RELA (p65) and thus inhibit p65 signaling (75). Additionally, Erβ has been found to downregulate the unfolded protein response (UPR), which enables the survival of cancer cell to endoplasmic reticulum stress induced by poor tumor vascularization (76). Furthermore, ERβ BC cell mitochondrial translocation inhibits TNBC proliferation of *vitro* and *in vivo* models via mitoERβ activation (77, 78). Finally, ERβ has been shown to inhibit epithelial to mesenchymal transition (EMT) and the invasiveness of TNBC *in vitro* (79) and inhibit metastatic TNBC phenotypes by suppressing TGFβ signaling through the regulation of cystatins (80). It is important to highlight that these alternate estrogen receptors can respond to systemic estrogen but, not to current endocrine treatments (81).

Hence, even in TNBC, estrogen can play a role in cancer progression, depending on levels of circulating estrogen, in which bacteria play a role and the cancer’s ability to respond *via* canonical or non-canonical receptors. In order to excrete estrogen from the body, the liver conjugates estrogen to glucuronic acid which can be then excreted in the bile. The GM has enhanced capacity to increase systemic estrogen levels by increasing enterohepatic circulation (82). GM bacteria can increase levels of systemic estrogen in two ways – first, by blocking the binding of estrogen to glucuronic acid, reducing its inactivation (83). Secondly, estrogens that are marked for excretion through the bile can be deconjugated by bacterial species expressing β-glucuronidases enabling their reuptake (84, 85).

GM bacteria can also metabolize phytoestrogens from dietary polyphenols, which are thought to modulate estrogen metabolism by reducing the systemic levels of circulation estrogen, as product of the inhibition of estrogen synthetase activity and reducing the bioavailability of ERs, for which they compete (86). In this regard. GM species such as *Eubacterium limosum* activate polyphenols (isoflavones and lignans) by demethylating their hydroxyl groups (87). Enterogenic lignan can then be transformed into bioactive enterolactone by other GM strains, such as members of genus *Eggerthella* (88). Interestingly, equol, the isoflavone derivative with the greatest estrogenic and antioxidant activity, is only found in one third to one half of humans (thus, only in those harboring equol-producing microbes) (89). The isoflavone daidzein found exclusively in soya beans and other legumes and can be converted to equol by several bacterial species including *Slackia*, *Lactobacillus*, *Paraeggerthella*, *Bifidobacterium* and *Eggerthella* sp. among others (89). While the effects of bacterially activated phytoestrogens on receptor positive BC are not yet agreed upon; a recent TNBC clinical trial, comprising 39 patients with invasive TNBC, established that a course of oral S‐equol inhibited proliferation of breast tumor cells, as measured by the cell proliferation marker Ki-67, with a 20% decrease in Ki‐67 expression in almost one third of patients (90).

##### 2.1.4.2 Short chain fatty acids

SCFAs are one of the main metabolites generated by the GM in the large intestine through anaerobic fermentation of indigestible dietary fiber and resistant starch (91). Among these, butyrate is the most important in relation to cancer, which is produced by Firmicutes (92). Cancer-driven histone deacetylase (HDAC) activity can lead to dysregulated epigenetic changes that silence tumor suppressor genes (TSG) facilitating malignant proliferation (93). Butyrate shows the most potent anti-cancer properties, including anti-inflammatory effects, suppression of angiogenesis, histone deacetylase (HDAC) inhibition which can reverse silencing of tumor suppressor genes (TSGs) and apoptosis induction in tumor cells by means of mitochondrial ROS production (94–97). A high fiber diet promotes the maintenance of butyrate-producing bacteria, making it cancer-protective. Conversely, the opposite effect is true; depletion of butyrate-producing bacteria may promote inflammation and tumorigenesis systemically (98). The receptors responsible for detecting SCFAs in TNBC are free the free fatty acid receptors (FFAR) 1 and 2 (99). *In vitro* TNBC studies have demonstrated that the activation of FFAR2 receptors increases the expression of adhesion proteins (E-cadherin) and inhibits MAPK signaling (*via* Hippo-Yap pathway inhibition), thus leading to a reduction of actin polymerization and cell invasiveness (100). This is supported by a study showing that the expression of both receptors was reduced in invasive breast carcinoma and metastatic TNBC tumors, relative to normal breast tissue (101).

##### 2.1.4.3 Bile acids metabolites

Lithocholic acid (LCA) is a bile acid metabolite that has been found to exert cancer protective effects. It is produced exclusively in the gut by a few species of anaerobic bacteria in the genus *Clostridium* from primary bile acids (102). Any bile acids found in breast tissue originate from the gut (103). LCA anticancer properties in relation to BC in general, include reductions in cancer cell proliferation and epithelial to mesenchymal cell transition inhibition acting *via* the G-protein-coupled bile acid receptor 1 (TGR5) that exerts downstream anti-inflammatory effects (104). It also inhibits angiogenesis by inhibiting vascular endothelial growth factor (VEGF) (105). LCA alters cellular metabolism by inducing glycolysis and increasing mitochondrial oxidative phosphorylation in BC cells that depend on the Warburg effect (104). The Warburg effect is a cancer specific effect, with increased glucose uptake and fermentation to lactate even in the presence of oxygen, proposed to be an evolutionary mechanism to sustain proliferative growth through the generation of essential biomolecules, by-products of glycolysis (94). BC patients have been found to have low levels of the LCA-producing gene 7α/β-hydroxysteroid dehydroxylase (baiH) detected in DNA extracted from stool samples in early cancer cases when compared with healthy controls (104, 106). In addition, microbiota-derived bile acids of GM origin accumulate in breast tumors and correlate with reduced proliferation (107). Other studies found that BC cells treated with LCA *in vitro* decreased the expression of nuclear factor-2 (NRF2) and increased the expression of Kelch-like ECH associating protein 1 (KEAP1), constitutive androstane receptor (CAR) and inducible nitric oxide synthase (iNOS) (108). All of which has been found to correlate with an improved survival rate of BC patients, except for TNBC (108). In this regard, studies investigating this in the different subtypes of TNBC, in particular, are thus needed.

##### 2.1.4.4 Polyamines

Polyamines are small polycationic molecules with a wide array of biological functions including gene regulation, stress resistance, cell proliferation and differentiation. These are mainly sourced from the GM, where they derive from bacterial amino-acid metabolism (109). Among these, Cadaverine is a biogenic amine derived from the decarboxylation of lysine and is used by bacteria to buffer the pH of their environment. It is synthesized by lysine decarboxylase (LdcC) and cadaverine A (CadA), enzymes found in species of the bacterial genera *Enterococcus*, *Enterobacter*, *Escherichia*, *Proteus*, *Streptococci*, and *Shigella* among other (110). Cadaverine has been shown to have tumor suppressor roles in breast cancer. Its antitumor effects have been proven in a TNBC murine model (grafted 4T1 tumor cells), with cadaverine found to reduce Epithelial to mesenchymal transition (EMT), an essential process driving tumor progression and metastasis, in which epithelial cells lose their features (cell polarity and cell–cell adhesion) and gain the invasive properties characteristic of mesenchymal stem cells. Consequently, cadaverine inhibited tumor growth, reduced cellular migration and invasion, ultimately reducing metastasis. In addition, cadaverine was also found capable of reducing BC invasion by inhibiting mesenchymal-to-epithelial (MET) transition (reverting mesenchymal tumor cells to a more epithelial like state) *via* the activation of trace amino acid (TAAR) cell receptors (111). This is supported by results from clinical trials (112)

##### 2.1.4.5 Indole derivatives

Indoles are bioactive products of the GM bacterial catabolism of tryptophan. Among these, 3-Indolepropionic acid (IPA), has been the subject of numerous studies due to its anticarcinogenic effects. IPA and other propionic acid species (PAs) derived from phenylalanine and tyrosine are synthesized through aromatic amino-acid transferases and phenyllactate dehydratase found in some species of the bacterial genera *Lactobacillus, Akkermansia, Clostridium* and *Peptostreptococci* (68, 113). In general, Indoles are also key interbacterial and GM-Host signaling molecules, act through steroid and xenobiotic receptors (AHRs and PXRs) (114). Indole derivatives prevent carcinogenesis by promoting gut homeostasis (upregulating tight junction, cell turnover, mucin and AMP secretion) (115) and modulate immune tolerance by shifting immune-cell polarization towards anti-inflammatory types. IPA is a free radical scavenger and a potent antioxidant, which prevents DNA damage in non-transformed cells exposed to multiple types of oxidative damage (113, 116). Furthermore, IPA and indoxyl-sulphate (IS) exert cytostatic effects in breast cancer cells (including TNBC) *in vitro* and *in vivo*, where they reduce cancer cell stemness, their EMT and proliferation (117). In a recent study, it was found that four new benzo[*f*]indole-4,9-dione derivatives reduce TNBC cell viability by ROS accumulation *in vitro* and exert cytotoxic effects on TNBC cells (MDA-MB 231) through the intrinsic apoptosis pathway – activation of the caspase 9 and Bax/Bcl-2 pathway (118).

### 2.2 The proximal microbiome and contribution to tumor development

Once thought to be sterile, it is now well established that the breast has its own unique microbial signature (119–123). Breast and milk microbiomes are related, which makes the milk microbiome a good predictor for Breast Microbiome (BM) composition in lactating women (124). Milk has been found to accommodate more than 360 species of bacteria, mainly from the *Actinobacteria*, *Bacteroidetes* and *Firmicutes* phyla (125). Due to various exogenous factors (dietary habits, geographic locations, lactating phase, and research methodologies) different studies report different microbial richness and composition at genus/species level. Despite this, *Staphylococcus* and *Streptococcus* have been present in 98.7 and 97.7% of the samples analyzed, respectively, and are considered as core genera, followed by lactic-acid specific bacteria (*Bifidobacterium* sp. and *Lactobacillus* sp.) (126).

Microbes residing in breast tissue have variable origins, as these can be sourced from different body-parts, through different interactions. In this regard, aerobes and facultative anaerobes from the skin and other epithelial surfaces may gain access to the breast through the nipple-areolar opening. For example, bacteria from the oral mucosa may gain access during breastfeeding and/or sexual contact (127). The growth and persistence of the infiltrated bacteria is sustained by the favourable conditions of breast tissue, featuring *(1)* nutrient-rich fatty tissue content, *(2)* diffuse ducts originating from the nipple; and *(3)* a widespread vasculature and lymphatic systems facilitating their movement (119).

Conversely, the microbial translocation of strictly anaerobic bacteria, which cannot survive in the presence of oxygen (e.g., *Bacteroides*, and some *Lactobacillus* and *Bifidobacterium* species) into breast tissue is far more complex and has been a topic of research and debate for decades. It has been suggested that some bacterial taxa may be translocated from the gut to the mammary tissue *via* an enteromammary pathway (128). This is supported by evidence from different studies showing that dendritic cells (DCs) can sample and engulf bacteria directly from the lumen (maintaining epithelial barrier integrity) (129) and transport them alive (for up to 60 h) to other lymphoid tissues (57). This was also supported by a seminal study of the origin of human breast milk bacteria. This study revealed that during the perinatal period there is a heightened bacterial translocation to the GALT, which is followed by bacterial colonization of the breast during the immediate postpartum. Here, it was also suggested that bacterial transport was mediated by DCs, which was supported by evidence showing that: (*i)* the majority of mononuclear cells in the milk originated from the GALT, (*ii)* staining showing bacteria-DCs co-localization and (*iii)* culturing of viable bacteria extracted from DCs purified form maternal milk and blood. Other preclinical studies have suggested GIT translocation of certain bacteria, such as bifidobacteria, to distal tumors (130).

Tumor colonization by bacteria is facilitated by the increased permeability of the tumor microenvironment (TME), with leaky vasculature due to rapid angiogenesis. Tumor selective growth of specific facultative and/or anaerobic bacterial strains is supported by the highly hypoxic and nutrient-rich necrotic tumor regions and its characteristic suppressed immune surveillance (131–134). Several recent studies have raised convincing evidence that associates the breast microbiome with cancer; however, its role in tumorigenesis is still a subject of active investigation, as it still unknown whether different microbial signatures are a cause or a product of tumorigenesis driven tissue remodeling (134, 135). Nevertheless, the role of intratumoral bacteria (including in TNBC) in tumor progression is now well-acknowledged and considered an enabling factor of “the hallmarks of cancer” due to their capacity to contribute to genome instability and mutation, and tumor-promoting inflammation (136).

#### 2.2.1 Unique TME signatures

In recent years various investigations have found characteristic microbial signatures associated with the breast cancer tumor microbiota (TM). In general, these studies point out that the microbial composition of breast tissue is dominated by bacteria from Proteobacteria, followed by Firmicutes, Actinobacteria and Bacteroidetes phyla, but BC tumor tissue features an altered composition in terms of abundance. In this regard, BC tumor tissue has an overrepresentation of Proteobacteria (a phylum usually associated with inflammation and disease), with higher abundances of Gammaproteobacteria, especially from the *Enterobacteriaceae* family and reduced abundance of Actinobacteria and Bacteroidetes. BC TM also has a higher abundance of pathobionts (pro-inflammatory) in the Firmicutes [e.g. *Staphylococcus* (137)*, Streptococcus pyogenes* (138)) and Actinobacteria *(Micrococcus* (139)*, Atopobium* (140)] phyla. Furthermore, the BC tumor microbiota showed an increase in abundance of taxa with known carcinogenic effects, such as those belonging to the *Fusobacterium* genus (121, 122, 141, 142). In fact, taxa from the *Enterobacteriaceae*, *Streptococcaceae*, *Staphylococcaceae* and *Micrococcaceae* families have been found in pancreatic cancer tumors (143) with *Staphylococcaceae* species in particular, also present in higher abundance within the tumor microbiota of prostate cancer (144). Likewise, in an ovarian cancer microbiota study, the Gammaproteobacteria *Shewanella* sp. was detected in 91% of cancer tissue samples (145). On the contrary, bacterial taxa with known anti-carcinogenic effects, such as species from the Bacteroidetes phyla and *Lactococcus*, and *Streptococcus* genera, were less abundant in BC TM (146).

For TNBC in particular, the studies described in **Table 1**, list the microbial signatures found to be enriched in the TNBC TME for each study. The lack of congruity between studies, reflects the lack of defined protocols for the study of breast/tumor microbiomes, wherein, a myriad of variables that can influence the outcomes/results. For example, differences in sample source (fresh-frozen/FFPE, ethnicity/geography), experimental protocols (collection: surgery/biopsy, DNA extraction, hybridization/sequencing), and bioinformatic workflows (146). Nevertheless, these studies offer insights to relevant features that are particular to the TNBC tumor microbiota. First, the TM of TNBC has the least taxonomic diversity among all BC types (148). Second, TNBC has higher abundance of *Aggregatibacter* and *Caulobacter* (147, 149) which are biofilm forming bacterial strains that have been previously associated with localised periodontitis and endocarditis (150, 151). Furthermore, these studies found that the microbial profiles found in tumor-adjacent non-cancerous (matched) tissue had a higher abundance of pathobionts and were more similar to the TM than those found in non-matched healthy tissue controls. This indicates that the TME resident tumor microbiota can extend to surrounding tissues and/or that microbial profiles found in the tumor pre-existed tumor formation, which would suggest that these microbes have an active role in tumorigenesis (148). This can be supported by recent evidence from a study involving seven different cancer types and over 1500 FFPE tumor samples, where intratumoral bacteria were found to localize within the tumor and immune cells (152).

**Table 1:** Description of studies of the TNBC microbiota.

Authors	Banerjee et al., 2015	Banerjee et al., 2021	Tzeng et al., 2021
Year	2015	2021	2021
Patient stratification	Breast cancer receptor type	Breast cancer receptor type, Tumor grade and stage, primary site of the tumor, response to treatment, survival, and disease-free time post treatment.	Breast cancer stage, grade, subtype, receptor type, and lymph node status.
No of TNBC samples	100	100	30
Non-cancer breast tissue	17 matched + 20 non-matched	20 matched + 68 non-matched	87 non-matched + 175 matched
Sample type	FFPE breast tumors + matched/non-matched controls	FFPE breast tumors + matched/non-matched controls	Freshly frozen breast tissue
Taxa enriched in TM *vs.* matched controls	Actinomycetaceae, Caulobacteriaceae, Sphingobacteriaceae, Enterobacteriaceae, Prevotellaceae, Brucellaceae, Bacillaceae, Peptostreptococcaceae, Flavobacteriaceae	Actinomyces, Bartonella, Brevundimonas, Coxiella, Mobiluncus, Mycobacterium, Rickettsia, Sphingomonas	Azomonas, Alkanindiges, Caulobacter, Proteus, Brevibacillus, Kocuria, Parasediminibacterium
Reference	(147)	(148, 149)	(146)

The TM of TNBC has the least taxonomic diversity among all BC types.

All these studies are merely taking a snapshot of the bacteria present at the time of sampling. The bacterial load is of extremely low biomass relative to the TME or the GM for that matter and issues of contamination and sampling techniques are important confounding factors. While our lab and others to improve the relevant methodology (153–156), bacteria at the scene cannot be pinned down to having a causative effect in oncogenesis or are merely bystanders having found a niche where they can survive (135).

#### 2.2.2 Protective effects

In a eubiotic state, the breast microbiota produces metabolites that may confer protection from pathogens, boost immune responses and inhibit tumorigenesis. Across different TNBC-specific studies, a higher abundance of BM commensals (e.g. *Streptococcus* sp.) that synthesize cadaverine, a known BC tumor suppressor, has been found to correlate with healthy breast tissue (111). A different study highlighted that butyrate-producing strains (e.g. *Odoribacter* sp.), recognised for their anti-inflammatory effects, were absent in tumor tissues (92, 146). Additionally, several breast tissue resident commensals, such as *Staphylococci* sp., *Lactococcus* sp., and *Streptococcus* sp. produce lantibiotics and other bacteriocins, preventing the potential growth of pathogenic strains that could trigger chronic inflammation. Beyond this, some bacteriocins have also been found to have selective cytotoxicity toward cancer cells (157). For example, *Lactobacillus salivarius* and *L. gasseri*, have been shown to clear mastitis infections during lactation when administered as a probiotic (158) and their excreted products (as cell-free supernatant) has been found to inhibit BC cells *in vitro* (159). Similarly, Bovicin HC5 from *Streptococcus bovis* HC5 has been shown to induce cell death in BC cell lines *in vitro* (160).

Furthermore, breast tissue resident bacteria also have the capability to activate the immune system *via* MAMPs. For example, the MAMP Flagellin (and the principal component of bacterial flagella) activates Toll-receptor 5 (TLR5). This is a PRR recently found to be specifically expressed in the ductal epithelium of normal breast tissues and circulating immature dendritic cells induces the secretion of pro-inflammatory cytokines and chemokines, increases tumor necrosis and neutrophil infiltration, inhibiting cell proliferation and anchorage-independent growth in mouse xenografts of human BC cells (161). In this context, *Lactococcus lactis* has been shown to recruit and activate natural killer cells (the main innate immunity cytotoxic effector cells toward cancer cells) (122). This has been confirmed in a clinical trial where *Lactococcus* abundance positively correlated with the number of NK cells recruited (162). Finally, healthy breast tissue is known to harbor bacterial strains, such as species of *Lactococcus* and *Streptococcus*, genera known to produce antioxidants and ROS scavengers (e.g. indoles, IPA) that neutralize free radicals and reduce oxidative damage, preventing oncogenesis (119, 163). Supporting all the protective benefits to breast health are extensive clinical studies and meta-analyses correlating the use of antibiotics with a moderately increased risk of BC (164–166).

#### 2.2.3 Microbial contributions to oncogenesis and tumor growth

Some bacterial signatures can induce cellular and immunomodulatory changes that promote oncogenesis, tumor growth and metastasis. Here these are referred to as Pathobionts. Now, while some of these microbes may not be a pathogen in other body parts they behave as such in this specific context, as the capacity of any microbe to act as a pathogen is context dependent, on location and state of immune activation. Some of these effects are listed in **Table 2**.

**Table 2:** Microbial contributions to oncogenesis and tumor growth.

Effects	Mechanisms
Genome damage	Some pathobionts, such as *E. coli* and *S. epidermidis* cultured from breast tissue has been shown to induce DNA double-strand breaks in HeLa cells *in vitro* (122).
Pro-inflammatory response	Dysbiosis of the BM disrupts the local homeostatic levels of MAMPs and increases the levels of ROS. This triggers the expression of pro-inflammatory cytokines and the release of DAMPs, which drives a positive feedback loop towards a chronic pathogenic inflammatory response, promoting tumorigenesis or enhancing pre-existing tumor growth (167).
Modulate immune response and survival	1) TLR signaling: In all types of BC, TLR (microbial sensing) receptors are significantly altered. BC features upregulated TLR4 receptors, which stimulate pro-inflammatory, pro-survival pathways (e.g., NF-κB) (146, 168, 169). In this regard, bacterial MAMPs bind to and modulate TLR4. For example, LPS binds to TLR4+ monocytes and promote their differentiation to pro-tumorigenic M2 macrophages (170, 171). 2) Immune cell recruitment: In healthy breast tissue, the abundance of certain bacterial strains, such as *Streptococcus, Propionibacterium*, *Staphylococcus*, and *Acinetobacter* positively correlate with that of tumor-targeting cytotoxic T-cell (e.g. CD8+), while their depletion contributes to an immunosuppressive TME typically found in BC (146). Conversely, the abundance of pathobionts, such as *F. nucleatum* inhibits the infiltration and effects of tumor-infiltrating lymphocytes (172) 3) Oncogene expression: Certain bacterial signatures can potentially regulate oncogene expression. For example, the presence of Staphylococcus has been negatively correlated with tumor necrosis factor receptor-associated factor 4 (TRAF4) (146, 173).
Promote Metastasis	1) Intracellular Pathobiont influence gene expression: Certain pathobionts, such as *Fusobacterium nucleatum* invade cancer cells and induce their proliferation while effectively evading the immune system (174). In BC, *F. nucleatum* colonization accelerates tumor growth and metastasis (172). Similarly, the presence of *Haemophilus influenzae*, has been correlated with the expression of pro-tumorigenic pathways (142). 2) MAMPs: Increased levels of circulating MAMPs, such as LPS has been associated with BC metastasis by inducing a monocyte-mediated endothelial adhesion of circulating cancer cells (175) or by inducing the production of pro-metastatic neutrophil extracellular traps (NETs) (176). Lymphovascular invasion is associated with a reduced abundance of *Oblitimonas* in the TM (146).

### 2.3 Microbiome effects on cancer treatments

#### 2.3.1 Cancer immunotherapies

Conventionally, breast cancers were deemed to be ‘cold’ in terms of the immune response, in that they generally do not evoke a robust immune response thereby making them less likely to respond to immune checkpoint inhibitor (ICI) therapies. However, TNBC is the most immunogenic out of all the BC types (177). The response to ICI can vary significantly from person to person, with only a modest subset of patients displaying increased survival rates (178). In a phase 1b clinical trial assessing the antitumor activity of an anti-PD-L1 antibody in patients with locally advanced or metastatic breast cancer, the confirmed objective response rate (ORR) for BC was 3.0% and 5.2% for TNBC. Results from this study suggest that the expression of PD-L1 by tumor-infiltrating lymphocytes (TIL) may predict the response to ICI therapy in breast cancer, as patients with TILs expressing PD-L1 showed an increased ORR in the overall population (16.7% *vs.* 1.6%), an effect that was remarkable in the TNBC subgroup, with an ORR of 22.2% *vs.* 2.6% (179). This confirmed previous findings in TNBC where a 10% increase in TILs was significantly correlated with a reduced reoccurrence of distant tumors in patients undergoing trastuzumab treatment in a large clinical trial, which is the reason why TILs are used as a biomarker of prognostic outcome (180). TILS can be subdivided into: CD4^+^ antigen-presenting T-helpers (Th) CD4^+^ immunosuppressive T-regs, and cytotoxic CD8+ T-cells – involved in direct tumor cell killing (see **Figure 2**). While high prevalence CD8^+^ T-cells and Th1 cell correlate with better treatment outcomes, the presence of Tregs and Th2 cells are associated with a poorer prognosis (more in **figure 2**) (181). In this regard, certain bacterial species known to colonize TNBC tumors (e.g., *Fusobacterium nucleatum, and Staphylococcus aureus*) have been found to shift TIL populations and induce a tumor suppressing, pro-inflammatory (Type 1) immune response in melanoma. Bacterial peptides (MAMPs) produced by these species are recognised by MHC I and II in APCs and stimulates the production of pro-inflammatory cytokines, which in turn recruit cytotoxic cells and induce pro-inflammatory Th1 cells differentiation (see **Figure 2**) (182).

Ever-increasing evidence supports the GM’s role in modulating treatment and a toxic response to cancer therapy, with several recent studies precisely demonstrating the response to ICI treatments across numerous cancer types (183–193). Here, different studies list varied species and genera associated with positive treatment outcomes, pointing to the complexity of confounding factors and interactions between the patient, the environment and their microbiome. However, in general, these studies report higher response to therapy and overall survival in patients with diverse microbiome profiles (eubiosis), containing *Bifidobacterium, Akkermansia* and *Enterococcus*, among others. On the other hand, patients with a reduced microbiome diversity (dysbiosis) responded poorly to this therapy (186, 189). This was confirmed in GF murine models. Mice receiving FMT from responders improved their response to anti-PD-LI, while those who received an FMT from non-responders developed resistance (185, 194)..

Interestingly, some studies have shown that the therapy can induce detrimental changes to the microbiome that may influence the development of resistance (183). This highlights the relevance of managing the microbiome richness during the course of treatment (193). Immunotherapies can also lead to a wide range of inflammatory side effects, including colitis, thyroid dysfunction, pituitary inflammation, and inflammatory arthritis, among others (195, 196). The combination of low efficacy, high cost and the risk of side effects calls for the development of effective biomarkers, potentially through GM composition. However, to date, there are not sufficient data to support this for TNBC; and thus, more studies are required.

#### 2.3.2 Radiation therapy

Initial studies showed that cancer radiotherapy (RT) has fewer side effects on germ-free mice (197); further studies confirm that bacteria are responsible for the toxic effects of radiotherapy (198, 199). It was also shown that during macrophage polarization, cell metabolism is altered by shifting the balance between glucose utilization and fatty acid oxidation, influencing the immune response in the TME. Radiotherapy response or resistance is highly dependent on the TME, and this alteration may change the radio sensitivity of cancer cells. M1 macrophages are thought to enhance the radio-sensitivity of BC cells; however, M2 macrophages can activate radio-resistance through IL-4/IL-13-mediated STAT6 phosphorylation and M2 polarization (200). One study found that the vancomycin treatment enhanced the RT efficacy and that butyrate, a metabolite generated by vancomycin-depleted gut bacteria, abolished the vancomycin effect (201). This is another example of context-dependent effects of the microbiome, wherein butyrate, which is regarded as a beneficial metabolite in the context of oncogenesis and cancer progression, can negatively impact the outcome of a cancer therapy. In addition, IPA (see 1.4.4) was found to exert an RT-protective effect on mice (202). Patients who had a toxic reaction to RT were associated with an over-abundance of Clostridium, Rosesporium, and Phascolarctobacterium (203).

#### 2.3.3 Chemotherapy

For patients with TNBC that has spread to the lymph nodes and is more than 1 cm in size, the American Society of Clinical Oncology (ASCO) recommends neoadjuvant chemotherapy. A lack of alternative treatments beyond initial surgeries and radiotherapy to reduce the tumor load means that chemotherapy remains the mainstay of treatment options. Common drugs include platinums, capecitabine doxorubicin, cyclophosphamide, paclitaxel, methotrexate, and fluorouracil (5-FU).

The microbiome affects many of these drugs not only in their efficacy but also in their level of toxic side effects. For platinums to work correctly, they must induce double-stranded DNA breaks, and the microbial production of ROS promote these mechanisms (204). A study in germ-free mice found that their cisplatin antitumor activity could be restored with the introduction of *L*. *acidophilus* (205).

A study from our lab examined the effects of bacterial species identified in breast cancer patient tumor samples on thirty standard chemotherapies *in vitro.* Results demonstrated an increase in the toxicity of six chemotherapeutic drugs, a decrease in nine, including doxorubicin and gemcitabine, and no effect in the remaining 15, with different bacterial species producing different effects (206). This was also verified in an *in vivo* mouse tumor model, with inhibitory effects on gemcitabine evident in tumors colonized by *E. coli*. Such findings suggest that response to therapy in BC tumors may be improved by microbiome modulation, and/or that the microbiome profile of a patient should be considered to inform treatment choice. To this end, our lab also demonstrated a proof of concept to utilize the microbiome signature of breast biopsies to infer the malignancy status of the tissue (123). Alternatively, in the context of bacteriotherapy (see later), another study from our lab demonstrated the ability of the natural enzymolome of introduced bacteria to mediate local tumor therapy in a murine model *via* activation of multiple systemically administered chemotherapeutics (207).

In relation to TNBC, there is only one recent study which looked at the GM of 30 patients with TNBC, identifying *Bacteroides* and *Ruminococcaceae* as taxa more abundant in TNBC patients who achieved a complete pathological response (pCR) after treatment with neoadjuvant chemotherapy compared with those who did not achieve a pCR. Patients with a partial response had higher quantities of *Bacteroides caccae* than those without any response (208). Resistance to the nucleoside analogue gemcitabine was found to be induced by intracellular gammaproteobacteria in a murine model, with treatment efficacy restored through antibiotic ablation of the bacteria (209).

## 3 Bacteria as TNBC theranostics

**Figure 3:**
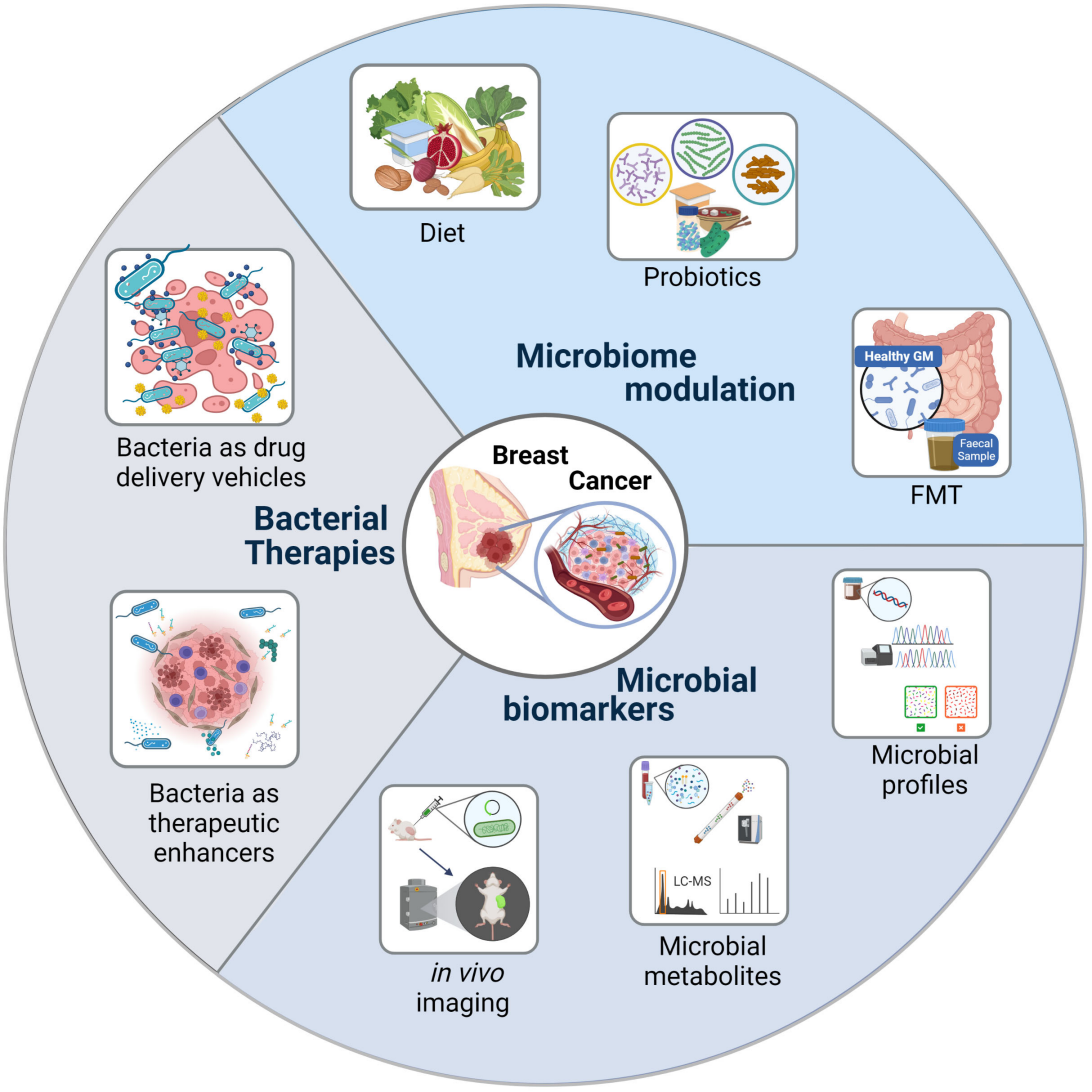
Bacteria as Cancer Theranostics. Harnessing the potentials of the microbiome to treat/prevent triple negative breast cancer.

The potentials of our microbiome can be harnessed to treat/prevent TNBC through therapeutic/life-style interventions that modulate our microbiome composition. Additionally, these commensals or their by-products can be used to develop early TNBC detection systems or to build bacterial based therapies. See **Figure 3**.

### 3.1 Dietary changes to reduce the risk of cancer

Prevention is better than cure; therefore, maintaining eubiosis through diet is extremely important. A western diet rich in fat, sugar and low in fiber combined with a sedentary lifestyle has been associated with a higher risk of BC (210). Obesity and gut dysbiosis go hand in hand, with both being associated with chronic inflammation, increased risk of developing BC and failure of cancer immunotherapy (211). In a mouse model of TNBC where obesity was induced *via* a western diet and compared with lean mice, tumor volumes throughout the experiment were significantly higher in obese animals, as expected, with an association between obesity and enhanced TNBC growth, with significant loss of diversity in the GM, showing a decrease in Bacteroides species, particularly *Alistipes* (211). Conflicting findings suggest that *Alistipes* exerts beneficial effects in colitis, has been associated with the increased efficacy of ICI and general activation of innate immunity whereas it has been found to be pathogenic in colorectal cancer (212).

Diet is crucially important in the interplay between the gut microbiome and estrogen metabolism, thereby affecting breast cancer metastasis depending to various degrees on breast cancer type, as a western diet is associated with increased levels of β-glucuronidase feeding estrogens back into the bloodstream. Low fiber has a compounding effect with a reduction in SCFAs such as butyrate which can help protect gut barrier maintenance and reduce inflammation, thereby adding to the pro-tumorigenic effect (213). Increased levels of inflammatory proteins systemically increase insulin resistance and leptin levels, both of which promote carcinogenesis (214). A reduction in adiponectin from adipose tissue contributed to insulin resistance and increased insulin-like growth factor 1 (IGF-1) levels, which can elicit increased cell proliferation (215).

Conversely, a diet high in fiber reduces β-glucuronidase expression, lowers systemic estrogen and increases sex hormone-binding globulin (SGBH) levels with fecal excretion of estrogen (213). A high fiber diet can also drive increased intestinal alkaline phosphatase production, essential for gut barrier integrity (216). Butyrate is also known to inhibit histone deacetylase and tumor progression (217). Retinoic acid derived from vitamin A combined with trichostatin (a synthetic HDACi) has been found to increase the HDAC inhibitory effect in murine xenograft models of BC (218). A diet rich in fiber and polyphenols has been found to enhance the BC survival rate (219, 220).

### 3.2 Probiotics

Probiotics can inhibit pathogenic bacteria colonization, help maintain eubiosis, promote gut barrier maintenance, reduce gut and systemic inflammation, and enhance immune and treatment response to BC (221–223). An *in vitro* study using the BC MCF-7 cell line found significant inhibition of cell proliferation, increased levels of apoptosis, and cell cycle arrest when using live, heat-killed cells (HKC) or cytoplasmic fractions (CF) of *E. fecalis* and *S. hominis* isolated from the breast milk of healthy women (224). Oral supplementation with a probiotic containing *Lactobacillus reuteri* alone was sufficient to inhibit BC tumorigenesis in murine models genetically predisposed to neoplasia and fed a cancer-promoting western diet through microbially-initiated CD4+/CD25+ lymphocytes (225). Moreover, researchers have found that oral administration of *L. acidophilus* can modulate immune responses *via* stimulation of IL-12 and promotion of Th1 production, a potent activator of NK cells, in a murine xenograft model of a breast adenocarcinoma (226). A further *in vivo* study showed that drinking probiotics containing *Lactobacillus helveticus* increased IL-10 and decreased IL-6 production in mice, which is vital in BC inhibition (227). A Japanese population-based case-control study involving 306 cases of BC and 662 controls found that regular consumption of probiotics containing *Lactobacillus casei* was inversely associated with BC incidence (228). Probiotics have also been found as an alternative to antibiotic treatment in cases of mastitis during breast feeding (158, 229, 230).

### 3.3 Fecal microbial transplantation

The most radical yet efficient means to modify the gut microbiome involves fecal microbial transplantation (FMT), a black-box approach, whereby the underlying mechanisms do not need to be fully understood for efficacy to be evaluated. The entire GM from a healthy donor, usually an exceptional responder to the treatment being applied, is transplanted into a recipient undergoing the same treatment. This technique has successfully reversed resistance to ICI treatment in two recent metastatic melanoma trials, associated with an increased abundance of Ruminococcaceae and Bifidobacteriaceae (231, 232). Besides FMT, where the entire donor GM is transplanted, other approaches focus on transplanting coalitions of bacteria (233) or even components of strain-specific bacteria in the case of *Enterococcus gallinarum* flagellin have been used (234).

Microbial signatures associated with reduced treatment-related toxicity can be taken advantage of using FMT or combinations of specific bacteria in all types of cancer treatments. FMT has been found to alleviate undesirable harmful effects of 5-fluorouracil-based chemotherapy in murine models of colorectal cancer (235). In pre-clinical models, FMT and indole 3-propionic acid have been observed to reduce radiation-associated toxicity (236, 237). Regarding BC in general and, more specifically, TNBC, these studies still need to be conducted.

### 3.4 Biomarkers of treatment efficacy/prognostic biomarkers of response to treatment

One recent study found the GM composition at BC diagnosis can serve as a prognostic marker. That α diversity was not predictive of favorable BC prognosis or side effects, and β diversity of the GM was associated with tumor grading but not BC subtype. Furthermore, the relative dominance of *C. bolteae, C. asparagiforme*, and *B. uniformis* in stool samples was associated with axillary lymph node invasion (238). It is now evident that the GM can affect all types of cancer treatments. This implies that bacteria associated with positive treatment outcomes can be used as biomarkers of treatment efficacy and toxicity, allowing for the much-touted advent of precise, tailored, personalised treatments (239–241).

### 3.5 Enhance treatment efficacy

Timing is everything or could play a role in chemotherapeutic treatment; one study using metronomic chemotherapy of capecitabine which is lower doses at more frequent intervals, showed promising results with reduced toxic side effects and less drastic changes to GM diversity. They found *Blautia obeum* to be associated with significantly prolonged PFS and significantly progression-free survival (PFS) with the occurrence of the *Slackia* genus (242). While chemotherapy can change the bacterial diversity, specific microbiome composition can, in turn, modify the efficacy of chemotherapy. Therefore, it is reasonable that particular probiotic concoctions could be administered adjunctively during chemotherapeutic treatment to augment effectiveness (221).

Oral administration of *Bifidobacterium longum* RAPO and ICI enhanced anti-PD-1 efficacy in preclinical murine models of TNBC (243). CRISPR-based phage therapy has recently been deployed to selectively wipe out nosocomial *Clostridioides difficile* infections, which are very difficult to treat without causing massive collateral damage to the patient’s beneficial GM (244). This approach used in TNBC treatment, combined with restoring lost valuable members of the GM, could synergistically rejuvenate patient GM functions and pave the way for establishing the best possible microbial amalgam for positive treatment outcomes. This approach has successfully been applied in murine models of atherosclerosis, with informed modification of the GM using small peptides to remodel the GM from that of one associated with a western diet to that of a low-fat-diet, resulting in reduced atherosclerotic plaques and a lowered pro-inflammatory cytokine profile (245).

### 3.6 Bacteriotherapy

In 1813, Vautier observed that cancer patients infected with *Clostridium perfringens* who developed gas gangrene appeared to be cured of cancer (246). Subsequently, bacterial infections and their effects on cancer were observed over 150 years ago by two German physicians W. Busch in 1868, and F. Fehleisen in 1882 (247, 248), who both independently found improvements in the condition of their patient’s symptoms, after a *Streptococcus pyogenes* infection. The first attempt to utilize bacteria as a cancer treatment followed shortly after, in 1893, when Dr William Coley combined the lytic compounds of *S. pyogenes* and *Serratia marcescens* and injected the mixture named Coley’s toxin into the tumor tissue with partial success in some patients (249).

There is a tricky balance when using bacteria in any therapy to get an effective therapeutic dose without toxic side effects on normal tissues. The advantage of using bacteria to deliver a genetic therapeutic cargo is their natural tumor tropism when systemically administered with high levels of local replication and the ability to persist within the TME. Furthermore, invasive species can invade tumor cells and, upon bacterial lysis, deliver a therapeutic DNA payload in the form of a bacterial plasmid which can be expressed directly in the tumor cell (250). Various payloads can allow for the direct killing of tumor cells or immunomodulation of the host immune system, increasing tumor antigenicity and promoting clearance by the immune system (251). Non-pathogenic Salmonella engineered with a quorum-sensing (QS) switch naturally hone to tumors and only express their therapeutic payload specifically within the TME once they have reached a particular critical density, thereby destroying cancerous tissue only (252). In preclinical models, activation of TLR5 by *S. typhimurium* flagellin in BC cells activates innate pro-inflammatory response for effective anti-tumor clearing (253). Bacteria can also be engineered to produce intrinsic bacterial biomolecules with known tumoricidal effects, some of which have been proven effective in human breast cancer cell lines (see **Supplementary Table 1**). These biomolecules include bacterial peptides, bacteriocin compounds, enzymes, or toxins.

Despite the large potential of bacteria-based mediated cancer treatments, the risks of adverse unmanageable side effects have tempered their use. Currently, the BCG vaccine is the standard for treating patients suffering from the Non-muscle invasive bladder cancer (254). 50 to 70% of patients have a positive outcome, with approximately 5% suffering adverse effects, including sepsis (255). Clinical trials with positive results include intra-tumoral administration with spores of the attenuated strain of *Clostridium novyi* (*C. novyi-*NT) to treat one patient suffering from advanced leiomyosarcoma, with promising results (256). Attenuated *L. monocytogenes* have also been found to be safe and effective in treating patients with advanced mesothelioma, lung, pancreatic, and ovarian cancers (257, 258). Pre-clinical studies using *Bifidobacterium* expressing cytosine deaminase (CD), which converted prodrug 5-fluorocytosine (5-FC) into chemotherapeutic agent (5-FU), was found to be effective in treating breast cancer when administered systemically in animal models (259–261).

## 4 Conclusion

The associations between TNBC and the microbiota are intricate, not yet fully understood, but undeniable. It is still not apparent whether changes in the GM and BM microbial composition are drivers of carcinogenesis or a response to tumor development. The effects are bidirectional, making it much more challenging to tease out these complex relationships. Furthermore, another issue is that these studies are just a snapshot in time. Bacteria have a short lifespan and quick generation time, changing over the course of the day with the body’s own circadian rhythms. Guilty microbes may have been and gone, whilst their effects rage on, especially in the case of DNA damage or epigenetic changes to the hosts’ genome, enabling cancer progression. Context is also critical concerning space and time; a bacterium in the gut may be beneficial but, when found in the TME, may exert the opposite effect. This also applies to distinct types of cancer. Bacterial metabolites can function as hormones, are highly pleiotropic and context-dependent, and can be produced distal to the tumor site while having systemic immune-modulatory effects.

Despite associations having been found in various microbiome-wide association studies, very little is known regarding the actual biochemical mechanisms. In the context of TNBC, even less is known, but lessons learned from studies involving other BC types are undoubtedly valuable. FMT also shows that even without the underlying mechanisms being fully understood, bacteria can still be used safely to improve treatment efficacy and reduce the toxic side effects of all types of cancer treatments currently used in TNBC.

More extensive clinical studies explicitly concerning TNBC are required to further elucidate the microbiome’s role and see past all of the confounding factors. Indeed, dietary changes should be taken on board to prevent cancer first, with probiotics (symbiont microbes) playing a role in returning the body to a state of normobiosis. Known associations can now be used as therapeutic and prognostic biomarkers, with enormous potential. The promise of completely personalized theranostics, however, is still a way off. Bacteriotherapy beyond FMT is further away, with plenty of evidence in *in vitro* and murine studies but lacking any clinical trials because of safety concerns. Genetic manipulation to reduce potential risks continues to make progress which, when matched to bacteria’s ability to selectively replicate within the TME, may in time overcome treatment hesitancy.

Indeed, monitoring of TNBC patients’ microbiomes may become standard practice where feasible, with modulation to a more beneficial state where possible. Altering the microbiome has potential to be the most unintrusive and safe means to change the body’s metabolism, improve treatment efficacy and reduce toxic side effects. Large-scale profiling of the GM and the associated metabolome of TNBC patients relative to that of healthy individuals will allow the development of theranostic biomarkers and treatments to improve the clinical prognosis and quality of life for TNBC patients.

## Author contributions

CD and YFB wrote the manuscript, CD prepared the tables and YFB the figures. MT revised the manuscript. All authors contributed to the article and approved the submitted version.

## Funding

The authors wish to acknowledge support relevant to this manuscript from Science Foundation Ireland (18/SP/3522) and Breakthrough Cancer Research, as part of the Precision Oncology Ireland consortium.

## Conflict of interest

The authors declare that the research was conducted in the absence of any commercial or financial relationships that could be construed as a potential conflict of interest.

## Publisher’s note

All claims expressed in this article are solely those of the authors and do not necessarily represent those of their affiliated organizations, or those of the publisher, the editors and the reviewers. Any product that may be evaluated in this article, or claim that may be made by its manufacturer, is not guaranteed or endorsed by the publisher.
