# Supplementary material for: Understanding and harnessing triple-negative breast cancer-related microbiota in oncology
Source: Front Oncol. 2022 Nov 24;12:1020121. doi: 10.3389/fonc.2022.1020121 (PMC9730816; doi:10.3389/fonc.2022.1020121)
Supplement: Supplementary file 1 [file Table_1.docx]

**Supplementary Table 1.** Bacterial Virulence Factors with anti-cancer properties against Breast Cancer Cell Lines

| **Name** | **Parent strain** | **Type of Biomolecule** | **BC cell lines tested** | **Significant notes and details** | **Ref.** |
| --- | --- | --- | --- | --- | --- |
| **Proximicins A–C** | *Verrucosispora sp.* | Non-ribosomal peptide | MCF-7 | Growth inhibition through induced upregulation of p53 and of the cyclin kinase inhibitor p21 | (1) |
| **Urukthapelstatin A** | *M. asporophorigenens* YM11-542 | Non-ribosomal cyclic thiopeptide | MCF-7 | Showed potent cytotoxic activity against a panel of human cancer cell lines. | (2) |
| **Entap** | *Enterococcus sp.* | Unclassified peptide | MDA-MB-231 | Interrupts cell proliferation, blocking cell division in G2, and inducing apoptosis | (3) |
| **Azurin** | *P. aeruginosa* | Copper-containing protein | MCF-7, MDA-MB-157 | Induces a p53-mediated cell cycle arrest in human breast cancer cells. | (4) |
| **p28** | *P. aeruginosa* | Part of azurin (amino acids 50-77) | MCF-7, ZR-75-1, T47D | A cell-penetrating peptide derived from azurin inhibits angiogenesis and tumour growth by inhibiting phosphorylation of VEGFR-2, FAK and Akt | (5) |
| **Pep27anal2** | *S. pneumonia* | Unclassified peptide | MCF-7 | Pep27anal2 induced apoptosis with minor membrane damage | (6) |
| **Bovicin HC5** | *S. bovis* | Lantibiotic - Class I bacteriocin | MCF-7 | Destabilises cell membranes through pore formation | (7) |
| **Nisin A** | *L. lactis* | Polycyclic bacteriocin | MCF-7 | Destabilises cell membranes through pore formation | (7) |
| **Laterosporulin 10** | *Brevibacillus sp.* SKDU10 | Defensin-like peptide | MCF-7 | LS10 displayed cytotoxicity against MCF-7, at below 10 μM concentration, | (8) |
| **Botulinum neurotoxin type A** | *C. botulinum* | Exotoxin | T47D, MDA-MB-231, MDA-MB-453 | Induces caspase-3 and -7 dependent apoptotic processes in the breast cancer cell lines | (9) |
| **Exotoxin A** | *P. aeruginosa* | Exotoxin | MCF-7, BT-20, CAMA-1, SKBR-3 | Inhibits the synthesis of protein via ADP-ribosylation of elongation factor-2 (EF-2) | (10) |
| **Exotoxin T** | *P. aeruginosa* | Exotoxin | MDA-MB-231, EMT6, 4T1 | This exotoxin has multiple cellular protein targets that have critical roles in survival, proliferation, metastasis, & angiogenesis in cancer, | (11) |
| **Hyaluronidases** | *S. pyogenes* | Enzyme | Hs578T, MDA-MB-231, MCF-7 | Recent studies have demonstrated that the addition of hyaluronidase to chemotherapeutic regimens could significantly improve efficacy | (12, 13) |
| **Colicin** | *E. coli* |  | MCF-7 | Pore-forming bacteriocins | (14) |

**REFERENCES**

1. Fiedler H-P, Bruntner C, Riedlinger J, Bull AT, Knutsen G, Goodfellow M, et al. Proximicin A, B and C, Novel Aminofuran Antibiotic and Anticancer Compounds Isolated from Marine Strains of the Actinomycete Verrucosispora†. The Journal of Antibiotics. 2008;61(3):158-63.

2. Matsuo Y, Kanoh K, Yamori T, Kasai H, Katsuta A, Adachi K, et al. Urukthapelstatin A, a novel cytotoxic substance from marine-derived Mechercharimyces asporophorigenens YM11-542. The Journal of antibiotics. 2007;60(4):251-5.

3. Karpiński TM, Adamczak A. Anticancer Activity of Bacterial Proteins and Peptides. Pharmaceutics. 2018;10(2):54.

4. Mohamed MS, Fattah SA, Mostafa HM. Azurin as antitumor protein and its effect on the cancer cell lines. Current Research Journal of Biological Sciences. 2010;2(6):396-401.

5. Yamada T, Mehta RR, Lekmine F, Christov K, King ML, Majumdar D, et al. A peptide fragment of azurin induces a p53-mediated cell cycle arrest in human breast cancer cells. Mol Cancer Ther. 2009;8(10):2947-58.

6. Lee DG, Hahm KS, Park Y, Kim HY, Lee W, Lim SC, et al. Functional and structural characteristics of anticancer peptide Pep27 analogues. Cancer Cell Int. 2005;5:21.

7. Paiva AD, de Oliveira MD, de Paula SO, Baracat-Pereira MC, Breukink E, Mantovani HC. Toxicity of bovicin HC5 against mammalian cell lines and the role of cholesterol in bacteriocin activity. Microbiology (Reading). 2012;158(Pt 11):2851-8.

8. Baindara P, Gautam A, Raghava GPS, Korpole S. Anticancer properties of a defensin like class IId bacteriocin Laterosporulin10. Scientific Reports. 2017;7(1):46541.

9. Bandala C, Cortés-Algara AL, Mejía-Barradas CM, Ilizaliturri-Flores I, Dominguez-Rubio R, Bazán-Méndez CI, et al. Botulinum neurotoxin type A inhibits synaptic vesicle 2 expression in breast cancer cell lines. Int J Clin Exp Pathol. 2015;8(7):8411-8.

10. Hemmati M, Tarighi P, Amoozadeh S, Farajollahi MM. Expression and purification of the recombinant pseudomonas exotoxin a conjugated to herceptin and its anti-proliferation effects on sk-br-3. Multidisciplinary Cancer Investigation. 2017;1:0-.

11. Goldufsky J, Wood S, Hajihossainlou B, Rehman T, Majdobeh O, Kaufman HL, et al. Pseudomonas aeruginosa exotoxin T induces potent cytotoxicity against a variety of murine and human cancer cell lines. Journal of medical microbiology. 2015;64(Pt 2):164.

12. Lee JH, Moore L, Kumar S, DeLucas L, Pritchard D, Ponnazhagan S, et al. In vitro studies on anti-cancer effect of Streptococcus pyogenes phage hyaluronidase (HylP) on breast cancer. Cancer Research. 2008;68(9_Supplement):1506-.

13. Lee JH, Moore LD, Kumar S, Pritchard DG, Ponnazhagan S, Deivanayagam C. Bacteriophage hyaluronidase effectively inhibits growth, migration and invasion by disrupting hyaluronan-mediated Erk1/2 activation and RhoA expression in human breast carcinoma cells. Cancer letters. 2010;298(2):238-49.

14. Chumchalová J, Šmarda J. Human tumor cells are selectively inhibited by colicins. Folia Microbiologica. 2003;48(1):111-5.
